# Thirty-seven-year trends in the prevalence, incidence, and prognosis of dementia in a Japanese community: the Hisayama study

**DOI:** 10.1186/s13195-025-01909-1

**Published:** 2025-12-29

**Authors:** Tomoyuki Ohara, Toshifumi Minohara, Taro Nakazawa, Emi Oishi, Yoshihiko Furuta, Satoko Sakata, Takanori Honda, Mao Shibata, Jun Hata, Tomohiro Nakao, Toshiharu Ninomiya

**Affiliations:** 1 Department of Neuropsychiatry, Graduate School of Medical Sciences, Kyushu University, Fukuoka, Japan; 2 Department of Epidemiology and Public Health, Graduate School of Medical Sciences, Kyushu University, Fukuoka, Japan; 3 Department of Medicine and Clinical Science, Graduate School of Medical Sciences, Kyushu University, Fukuoka, Japan; 4 Center for Cohort Studies, Graduate School of Medical Sciences, Kyushu University, Fukuoka, Japan; 5 Department of Psychosomatic Medicine, Graduate School of Medical Sciences, Kyushu University, Fukuoka, Japan; 6 Department of Health Care Administration and Management, Graduate School of Medical Sciences, Kyushu University, Fukuoka, Japan

**Keywords:** Dementia, Prevalence, Incidence, Survival rate, Mortality

## Abstract

**Background:**

Few population-based studies have investigated how the prevalence, incidence, and survival rate of dementia have changed since the 2010s in Asian communities. We investigated this issue using 37 years of epidemiological data in a Japanese community.

**Methods:**

Seven cross-sectional surveys of dementia were conducted among residents aged ≥ 65 years in a Japanese community in 1985, 1992, 1998, 2005, 2012, 2017, and 2022. We also established three cohorts in the residents aged ≥ 65 years without dementia in 1988 (*n* = 803), 2002 (*n* = 1,231), and 2012 (*n* = 1,519), each of which was followed for 10 years. Trends in the prevalence of dementia were tested using a logistic regression model. The age- and sex-adjusted incidence of dementia and survival rate after dementia onset were compared across cohorts using a Cox proportional hazards model.

**Results:**

The crude prevalence of dementia significantly increased from 1985 to 2012 (6.7% in 1985, 5.7% in 1992, 7.1% in 1998, 12.5% in 2005, and 17.9% in 2012, p for trend < 0.01), but then decreased significantly from 2012 to 2022 (15.8% in 2017 and 12.1% in 2022; p for trend < 0.01). A similar trend was observed after adjusting for age and sex. Moreover, the age- and sex-adjusted incidence of dementia increased significantly from the 1988 to the 2002 cohort (adjusted hazard ratio [aHR] 1.68, 95% confidence intervals [CI] = 1.38–2.06), but decreased significantly from the 2002 to the 2012 cohort (aHR = 0.60, 95% CI = 0.51–0.70). The age- and sex-adjusted 5-year survival rate after dementia onset increased significantly from the 1988 to the 2002 cohort (47.3% to 65.2%; *p* < 0.01), while no significant change was observed from the 2002 to the 2012 cohort (65.2% to 58.9%; *p* = 0.42).

**Conclusions:**

Decreasing trends in the prevalence and incidence of dementia were observed since 2012 in a Japanese community. The decline in the incidence of dementia may be due to the prevention and improved management of lifestyle-related diseases, such as hypertension and diabetes, as well as increased awareness and promotion of healthy lifestyle behaviors.

**Supplementary Information:**

The online version contains supplementary material available at 10.1186/s13195-025-01909-1.

## Background

With the aging of the global population, the number of individuals with dementia is expected to increase. Consequently, the medical, social, and economic burden associated with dementia remains a major public health concern [1]. 

Several epidemiological studies conducted in community-dwelling populations have reported an increasing trend in the prevalence of dementia [2–7]. In contrast, other epidemiological studies, primarily from Western countries, have suggested stable or declining trends in the prevalence of dementia [8–13]. Estimating the changes in the incidence and prognosis of dementia across time could help to resolve such discrepant findings. In addition, since dementia prevalence is determined by the balance between the incidence and survival rates after disease onset, such longitudinal estimation could improve our understanding of the underlying causes of shifts in dementia prevalence. Accordingly, multiple population-based and medical data-based prospective studies, mainly in Western populations, have investigated the changes in incidence and post-onset survival rates of dementia onset over time, although their findings have been inconsistent [2, 4, 6, 8, 12, 14–28]. Furthermore, secular trends in the prevalence, incidence, and prognosis of dementia have not been fully investigated in Asian communities, particularly using long-term survey data from a single community.

The Hisayama Study is an ongoing population-based cohort study of cardiovascular disease, other lifestyle-related diseases, and dementia conducted since 1961 [29]. Using the Hisayama Study data, we previously reported that dementia prevalence increased significantly in a Japanese community from 1985 to 2012, and that both increasing incidence of dementia and improvements in survival rates after onset likely contributed to this increasing trend [21]. To extend this line of investigation, we here investigated the prevalence and incidence of dementia, and the survival rate after dementia onset, in a community-based, older Japanese population using data from dementia surveys conducted by the Hisayama Study over a period of 37 years.

## Methods

### Study populations

The town of Hisayama is located in the Fukuoka metropolitan area in southern Japan. According to the national census, the age and occupational distributions and the nutrient intake of residents in this town have been almost identical to those of Japan for the past 60 years [29]. Since 1985, full-community surveys on dementia have been conducted every 5 to 7 years for Hisayama residents aged ≥ 65 years [21]. In 1985, 887 individuals (353 men and 534 women) among 938 residents in that age group participated in the survey (participation rate 94.6%)[30]. We performed similar screening surveys on 1,189 (475 men, 714 women) of 1,231 residents (participation rate 96.6%) in 1992; 1,437 (571 men, 866 women) of 1,442 residents (99.7%) in 1998; 1,566 (612 men, 954 women) of 1,711 residents (91.5%) in 2005; 1,906 (780 men, 1,126 women) of 2,036 residents (93.6%) in 2012; 2,202 (932 men, 1,270 women) of 2,340 residents (94.1%) in 2017; and 2,302 (979 men, 1,323 women) of 2,422 residents (95.0%) in 2022.

To clarify changes in the incidence of dementia and survival rates after dementia onset, we analyzed the data from a new 10-year follow-up cohort, along with data from the two cohorts (the 1988 and 2002 cohorts) reported in our previous paper (Fig. e-1)[21]. Briefly, among 837 residents (participation rate 91.8%) aged ≥ 65 years in the 1988 screening survey, 803 participants (313 men, 490 women) were enrolled in the 1988 cohort after excluding 34 individuals who had already developed dementia at baseline. Similarly, among 1,353 residents (83.2%) aged ≥ 65 years in the 2002 survey, 1,231 participants (529 men, 702 women) formed the 2002 cohort after excluding 122 participants with prevalent dementia. Finally, in the 2012 survey, among 1,906 participants aged ≥ 65 years (93.6%), 1,519 participants (654 men, 865 women) were enrolled as the 2012 cohort after excluding participants who did not consent to follow-up surveys (*n* = 44), those having prevalent dementia (*n* = 30), those lacking cognitive assessments (*n* = 2), and those with impaired consciousness or mental retardation (*n* = 2).

### Cross-sectional survey of dementia

We conducted a two-stage prevalence survey of dementia at each examination in the same manner [30]. In the first screening survey, we administered several neuropsychological tests: the Hasegawa Dementia Scale (HDS)[31], the HDS revised version (HDS-R)[31], and the Mini-Mental State Examination (MMSE) [32]. The HDS and HDS-R are commonly used in Japan and correlate well with the MMSE [30, 32]. When the test scores were lower than the cutoff points (22/32.5 for the HDS, 21/30 for the HDS-R and MMSE)[30], we carried out a second screening survey of dementia, including physical and neurologic examinations, interviews of the families and attending physicians, and review of the medical records [30]. Each participant was also asked to complete questionnaires regarding activities of daily living, sociodemographic status, and medical conditions [30]. 

### Follow-up survey of dementia

Each cohort was followed prospectively for 10 years. Details of the follow-up survey on dementia have been reported elsewhere [21, 29]. As reported previously[21], we used an established daily-monitoring system comprising the study team, local physicians, and members of the town’s Health Office to regularly collect information on new neurological events, including any cognitive decline and stroke. We also conducted regular health check-ups annually to identify incident cases of dementia. Postal and telephone surveys were performed for participants who did not undergo regular health check-ups or moved out of town. Moreover, to precisely detect dementia cases to the greatest extent possible, we conducted comprehensive neuropsychological screening for dementia in 1992, 1998, 2005, 2012, 2017, and 2022. Participants suspected of having new neurological symptoms, including dementia, were carefully evaluated for the presence or absence of dementia by an expert psychiatrist or a stroke physician of the study team. In addition, when a participant died, we collected and fully examined all the available medical information, including neuroimaging (CT/MRI), interviewed the family and attending physician of the deceased, and tried to obtain permission for autopsy from the family. Other than deceased cases, no participants were lost to follow-up in any cohort.

### Diagnosis of dementia

In the cross-sectional surveys, the diagnosis of dementia and its subtypes was made clinically based on the guidelines of the Diagnostic and Statistical Manual of Mental Disorders, third edition (DSM-III) [33] in 1985 and those of the DSM-III revised version (DSM-III-R)[34] in 1992, 1998, 2005, 2012, 2017, and 2022. We classified mixed-type dementia, i.e., co-occurrence of Alzheimer’s disease (AD) and vascular dementia (VaD), as other dementia in each survey.

In the follow-up surveys, dementia diagnosis was made based on the DSM-III-R guidelines [34]. Dementia subtypes were AD and VaD; AD was diagnosed based on the criteria of the National Institute of Neurological and Communicative Disorders and Stroke and the Alzheimer’s Disease and Related Disorders Association[35], and VaD based on the National Institute of Neurological Disorders and Stroke-Association Internationale pour la Recherche et l’Enseignement en Neurosciences criteria [36]. We used both clinical and neuroimaging and/or neuropathological findings to diagnose dementia subtypes, as reported previously [21]. We also counted mixed cases of AD and VaD as events in the analysis for each subtype. Each case of dementia was adjudicated by expert psychiatrists and stroke physicians.

### Risk-factor measurements

At the baseline survey of each cohort, a self-administered questionnaire concerning lifestyle—including smoking habits, alcohol drinking, educational status, medical history, and treatment of hypertension, diabetes mellitus, and hypercholesterolemia—was checked by trained interviewers. The mean of three measurements of blood pressure was used for the analysis. Hypertension was defined as blood pressure ≥ 140/90 mmHg or current use of antihypertensive agents. Diabetes mellitus was defined by fasting glucose concentrations ≥ 7.0 mmol/L, 2-hour postload glucose or postprandial glucose concentrations ≥ 11.1 mmol/L, and/or use of glucose-lowering agents (oral hypoglycemic agents and/or insulin). Hemoglobin A1c (HbA1c) levels were corrected to the National Glycohemoglobin Standardization Program equivalent value calculated as HbA1c (%) = 1.02 × HbA1c (Japan Diabetes Society) (%) + 0.25%[37]. Serum glycated albumin (GA) was measured enzymatically using an albumin-specific proteinase, ketoamine oxidase, and an albumin assay reagent (Lucica GA-L; Asahi Kasei Pharma, Tokyo). Serum total cholesterol levels were measured enzymatically, and hypercholesterolemia was defined as serum total cholesterol ≥ 5.69 mmol/L or use of lipid-modifying agents. History of stroke was defined based on all clinical data available in the Hisayama Study. Electrocardiogram abnormalities were defined as left ventricular hypertrophy (Minnesota Code 3 − 1), ST depression (4 − 1, 2, or 3), or atrial fibrillation (8 − 3). We measured body height and weight in light clothing without shoes and calculated the body mass index (BMI) (kg/m^2^). Leanness and obesity were defined as BMI < 18.5 kg/m^2^ and BMI ≥ 25 kg/m^2^, respectively. Low educational level was defined as ≤ 9 years of formal education. We classified alcohol intake and smoking habits as being either current habitual or not. Regular exercise was defined as engaging in sports or other forms of exercise (excluding recreational walking) at least three times a week during leisure time. Daily physical activity levels were reported for common occupational/domestic activities as follows: mostly sitting or lying down all day; mixed sitting; standing and walking; walking; and heavy labor. Sedentariness was defined as mostly sitting or lying down all day. Regarding the data for fiscal year 2022 (Table e-8), the dementia survey was conducted between 2022 and 2023, and the comprehensive health check-up was conducted in 2023; accordingly, the 2023 data were used.

### Statistical analysis

The age-standardized prevalence of dementia was estimated by the direct method using the WHO World Standard Population [38] with 5-year age groups. Trends in the crude and adjusted prevalence of dementia were estimated using a logistic regression model. The differences of crude and adjusted mean values and frequencies of risk factors among the three cohorts were estimated using a linear or logistic regression analysis, respectively. The incidence of dementia and its subtypes and all-cause mortality were calculated by the person-year method with adjustment for age and sex by the direct method with 5-year age groups. In this adjustment, the age and sex distribution in the 1988 cohort was used as a standard population. The age- and sex-adjusted incidence was compared between the two cohorts using a Cox proportional hazards model, and the adjusted hazard ratio (HR) with its 95% confidence interval (CI) was also estimated using a Cox model. Participants who developed dementia were followed-up for 5 years after disease onset or to the end of the follow-up period in each cohort, and survival curves were drawn using a Cox model with adjustment for age and sex. In each cohort, we also selected cases of dementia developed during the follow-up and age- and sex-matched control participants randomly selected from those without incident dementia and compared the 5-year mortality rate after dementia onset between these two groups. The heterogeneity in the influence of incident dementia on mortality between cohorts was tested by adding interaction terms to the relevant Cox model. All statistical analyses were performed with SAS (version 9.4; SAS Institute, Cary, NC). Two-sided *p* < 0.05 was considered statistically significant in all analyses.

### Data Availability

The datasets used in this study are not publicly available, because they contain confidential clinical data on the study participants. However, the data are available on reasonable request and with the permission of the principal investigator of the Hisayama Study, Toshiharu Ninomiya.

## Results

### Trends in the prevalence of dementia over time

Table e-1 shows demographic characteristics of participants in the seven cross-sectional surveys. The mean age of the participants increased from 74 years in 1985 to 77 years in 2022. From 1985 to 2005, women accounted for about 60% of total participants. Table 1 shows the prevalence of all-cause dementia and its subtypes in the seven surveys. The age-standardized prevalence of all-cause dementia and AD showed a significant increasing trend from 1985 to 2012 (both p for trend < 0.01), followed by a significant decreasing trend from 2012 to 2022 (both p for trend < 0.01). There was no significant trend in the age-standardized prevalence of VaD and other/unclassified dementia between 1985 and 2012 (p for trend = 0.59, p for trend = 0.15, respectively), while a significant decreasing trend was observed between 2012 and 2022 (p for trend < 0.01 for VaD, p for trend = 0.04 for other/unclassified dementia, respectively).

**Table 1:** Secular trends in the prevalence of dementia and its subtypes from 1985 to 2022

	Year of survey								
	1985 (*n* = 887)	1992 (*n* = 1,189)	1998 (*n* = 1,437)	2005 (*n* = 1,556)	2012 (*n* = 1,904)	2017 (*n* = 2,202)	2022 (*n* = 2,302)	p for trend (1985–2012)	p for trend (2012–2022)
All-cause dementia									
No. of cases	59	68	102	195	341	348	279		
Crude prevalence, %	6.7 (5.0–8.3)	5.7 (4.4–7.1)	7.1 (5.7–8.5)	12.5 (10.7–14.2)	17.9 (16.0–19.8)	15.8 (14.3–17.3)	12.1 (10.8–13.5)	< 0.01	< 0.01
Age-standardized prevalence, %	6.8 (4.8–8.8)	4.6 (3.4–5.8)	5.3 (4.2–6.4)	8.4 (7.1–9.7)	11.3 (10.0–12.7)	9.9 (8.6–11.1)	7.2 (6.1–8.2)	< 0.01	< 0.01
Alzheimer’s disease									
No. of cases	12	21	50	96	233	233	202		
Crude prevalence, %	1.4 (0.6–2.1)	1.8 (1.0–2.5)	3.5 (2.5–4.4)	6.1 (4.9–7.4)	12.2 (10.7–13.8)	10.6 (9.3–11.9)	8.8 (7.6–9.9)	< 0.01	< 0.01
Age-standardized prevalence, %	1.5 (0.5–2.5)	1.4 (0.7–2.0)	2.4 (1.7–3.1)	3.9 (3.1–4.8)	7.2 (6.1–8.2)	6.3 (5.3–7.3)	5.0 (4.1–5.9)	< 0.01	< 0.01
Vascular dementia									
No. of cases	21	22	25	51	58	34	33		
Crude prevalence, %	2.4 (1.4–3.4)	1.9 (1.1–2.6)	1.7 (1.1–2.4)	3.3 (2.4–4.2)	3.0 (2.3–3.8)	1.5 (1.0–2.1)	1.4 (0.9–1.9)	0.02	< 0.01
Age-standardized prevalence, %	2.4 (1.3–3.5)	1.6 (0.9–2.2)	1.5 (0.8–2.1)	2.4 (1.7–3.2)	2.4 (1.7–3.0)	1.3 (0.8–1.8)	1.0 (0.6–1.4)	0.59	< 0.01
Other/unclassified dementia									
No. of cases	26	25	27	48	50	81	44		
Crude prevalence, %	2.9 (1.8–4.1)	2.1 (1.3–2.9)	1.9 (1.2–2.6)	3.1 (2.2–3.9)	2.6 (1.9–3.4)	3.7 (2.9–4.5)	1.9 (1.4–2.5)	0.58	0.11
Age-standardized prevalence, %	2.9 (2.1–3.7)	1.7 (1.0–2.4)	1.4 (0.8–2.0)	2.0 (1.4–2.6)	1.8 (1.2–2.3)	2.2 (1.6–2.9)	1.2 (0.7–1.6)	0.15	0.04

Values in parentheses are the 95% confidence intervals

**Fig. 1:**
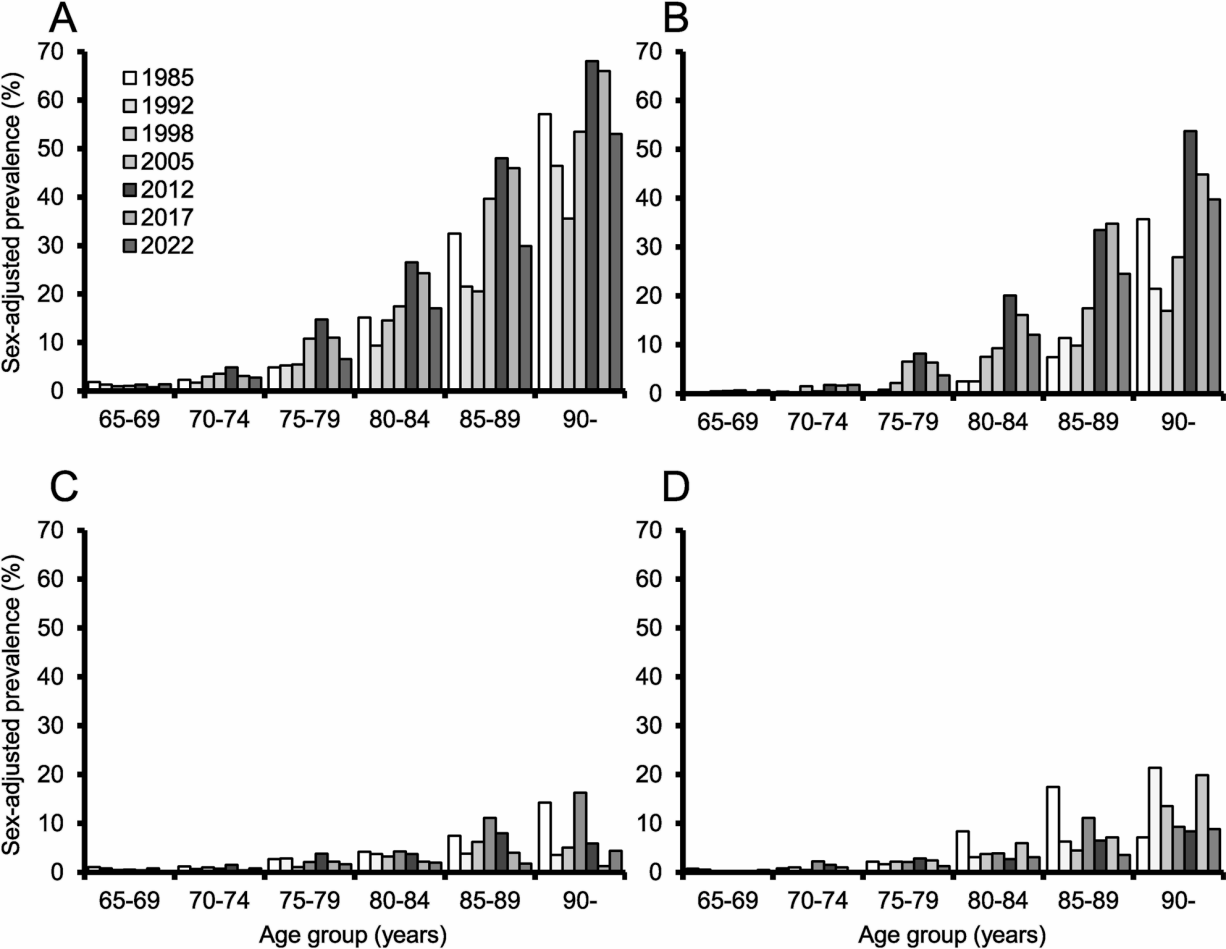
Secular trends in the sex-adjusted 5-year age-specific prevalence of dementia and its subtypes. **A** All-cause dementia. **B** Alzheimer’s disease. **C** Vascular dementia. **D** Other/unclassified dementia

Figure 1 demonstrates secular trends in the sex-adjusted prevalence of all-cause dementia and its subtypes by 5-year age groups. The sex-adjusted prevalence of all-cause dementia increased from 1985 to 2012 among participants aged ≥ 70 years (all p for trend < 0.05), followed by a significant decreasing trend from 2012 to 2022 among participants aged ≥ 75 years (all p for trend < 0.05). A similar significant trend was observed in AD among participants aged ≥ 75 years, with peak prevalence in 2012 (all p for trend < 0.05). There was no clear secular trend in the prevalence of VaD and other/unclassified dementia.

### Trends in the incidence of dementia over time

To investigate the incidence of dementia over time, we established three cohorts of dementia-free participants aged ≥ 65 years who underwent the baseline survey in 1988, 2002 or 2012 and were followed for 10 years (Fig. e-1). During the follow-up, 134 (40 men, 94 women), 334 (114 men, 220 women) and 290 (111 men, 179 women) individuals developed all-cause dementia in the 1988, 2002, and 2012 cohorts, respectively. Among these incident dementia cases, brain imaging and/or brain autopsy were used for evaluation in 130 (97.0%), 306 (91.6%), and 227 (78.3%) participants in the 1988, 2002, and 2012 cohorts, respectively. In addition, 7, 15, and 19 cases had mixed-type dementia (AD and VaD) in the 1988, 2002, and 2012 cohorts. These cases were counted as events in the analysis for each subtype. Finally, the respective numbers of patients with each subtype in the 1988, 2002 and 2012 cohorts were 73, 222, and 210 for AD; 48, 87, and 51 for VaD; and 20, 40, and 45 for other/unclassified dementia.

**Fig. 2:**
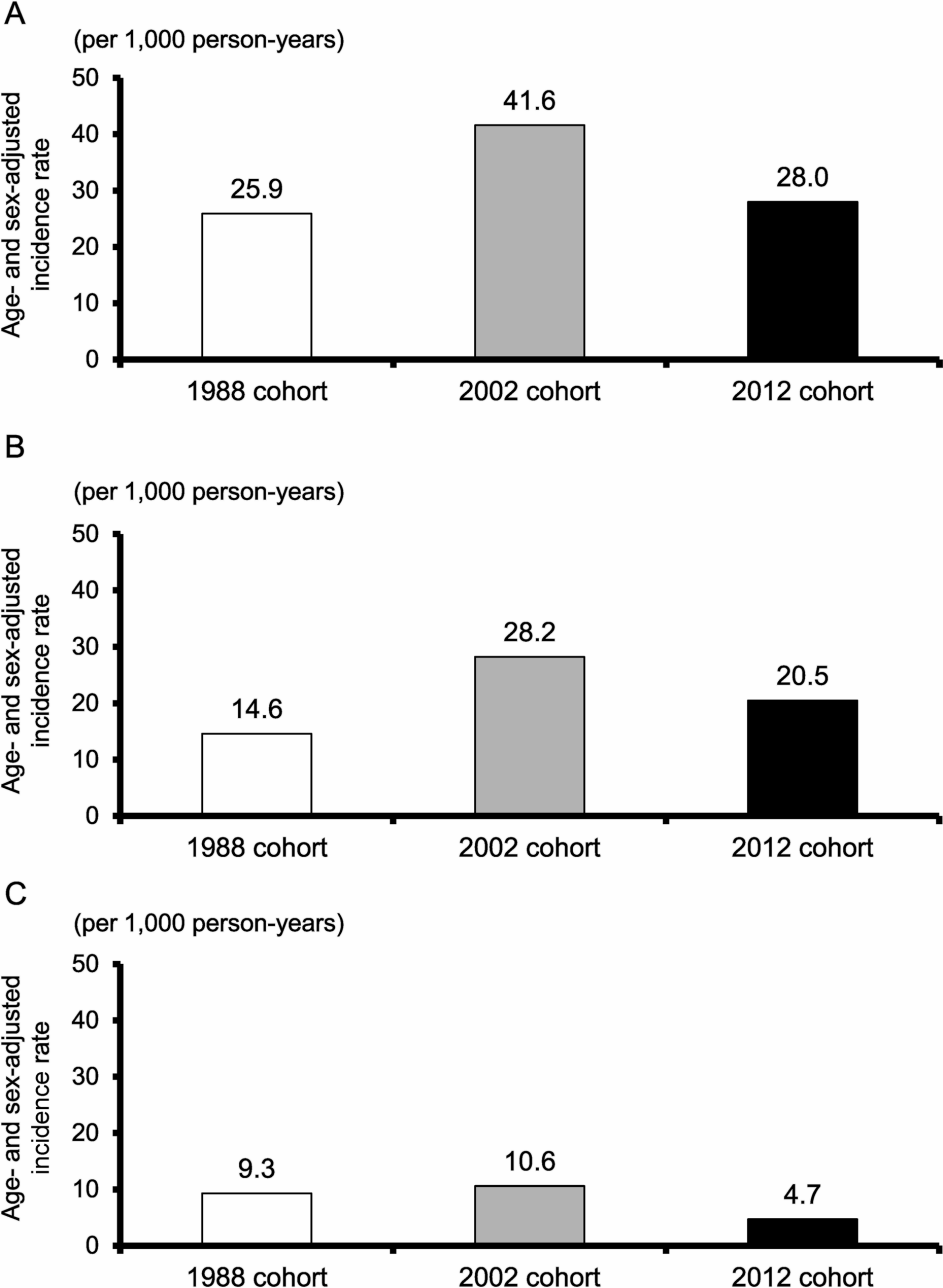
Comparison of the age- and sex-adjusted incidence of dementia among the three cohorts. **A** All-cause dementia. **B **Alzheimer’s disease. **C **Vascular dementia HR = hazard ratio; CI = confidence interval

Figure 2 compares the age- and sex-adjusted incidence of all-cause dementia and each subtype among the three cohorts. The incidence of all-cause dementia increased significantly from the 1988 to the 2002 cohort (HR 1.68; 95% CI 1.38–2.06; Table e-2) and then decreased significantly from the 2002 cohort to the 2012 cohort (HR 0.60; 95% CI 0.51–0.70; Table e-3). There was no significant difference in the mean age of onset of all-cause dementia between the 1988 and 2002 cohort (82.8 years for the 1988 cohort, 81.9 years for the 2002 cohort), but the mean onset age was significantly higher in the 2012 cohort than the 2002 cohort (81.9 vs. 83.0 years, respectively; *p* = 0.04). Similar significant trends were observed for AD. There was no significant change in the incidence of VaD from the 1988 to the 2002 cohort, but VaD incidence was significantly lower in the 2012 cohort than the 2002 cohort (HR 0.41; 95% CI 0.29–0.58; Table e-3). There was no significant change over time in the incidence of other/unclassified dementia. Tables e-2 and e-3 show the detailed data.

**Fig. 3:**
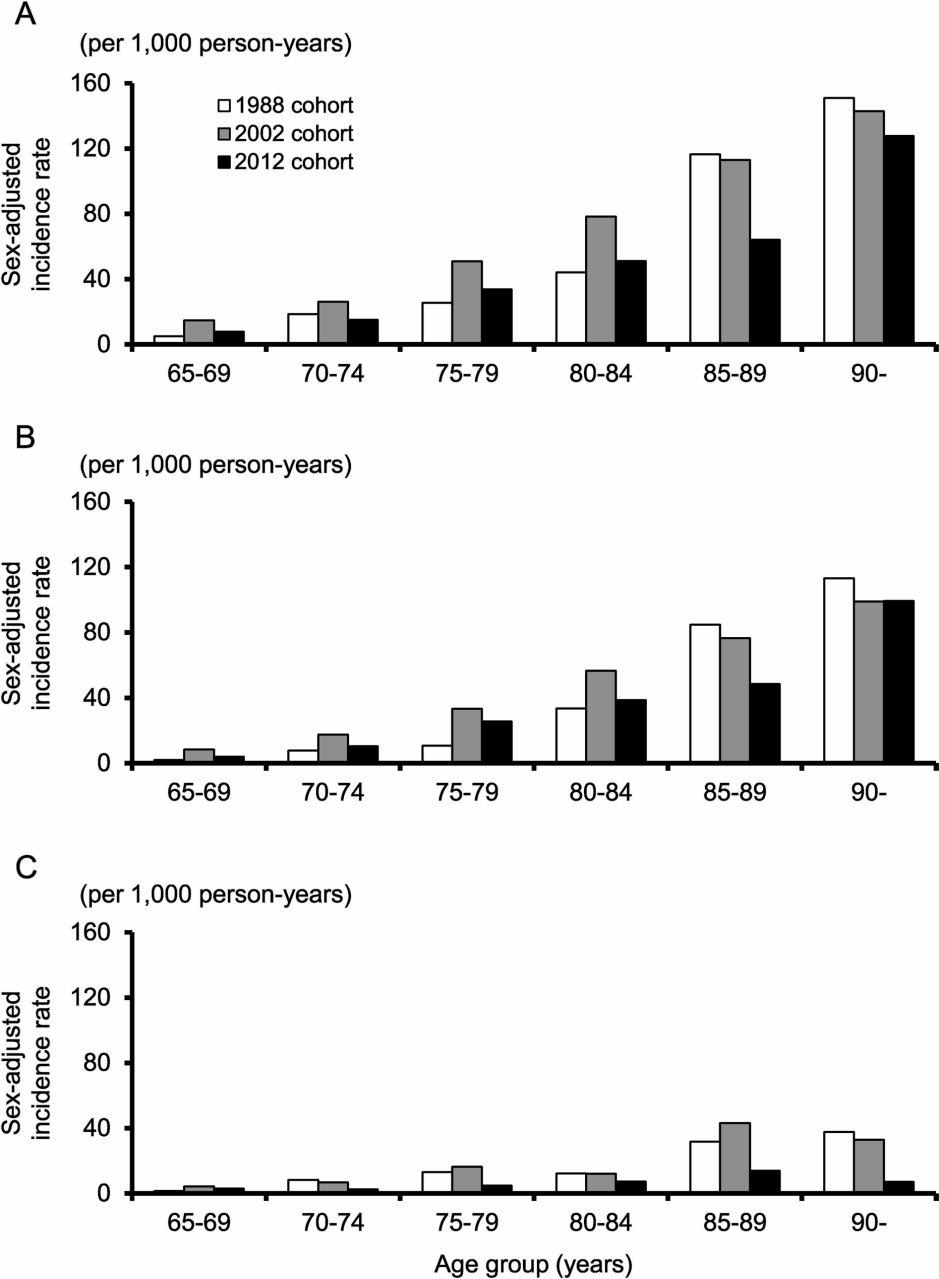
Comparison of the sex-adjusted 5-year age-specific incidence of dementia among the three cohorts. **A** All-cause dementia. **B** Alzheimer’s disease. **C** Vascular dementia

Figure 3 demonstrates the sex-adjusted incidence of all-cause dementia and each subtype in the three cohorts by 5-year age group. The incidence of all-cause dementia increased marginally/significantly in participants aged 65–84 years from the 1988 to the 2002 cohort, but marginally/significantly decreased in participants aged 65–89 years from the 2002 to the 2012 cohort. Similar trends were observed for the incidence of AD. The incidence of VaD did not change between the 1988 and the 2002 cohort, but marginally/significantly decreased in participants aged 70–89 years from the 2002 to the 2012 cohort.

To examine the factors related to changes in the incidence of dementia, the crude and age and sex-adjusted frequencies and mean values of risk factors at the baseline surveys were compared among the cohorts (Table e-4). The frequency of low education (≤ 9 years) continued to decrease across the three cohorts. Hypertension frequency was unchanged between the 1988 and 2002 cohorts, but significantly increased between the 2002 and 2012 cohorts. Meanwhile, systolic blood pressure significantly decreased over time, in correspondence with the increased use of antihypertensive agents. The frequency of obesity and diabetes mellitus increased from the 1988 to the 2002 cohort and then plateaued between the 2002 and 2012 cohorts. The frequency of leanness was lower in the 2002 cohort than in the 1988 cohort, whereas the proportion of use of glucose-lowering agents, especially oral hypoglycemic agents, increased with time. Among participants with diabetes mellitus, mean values of HbA1c were lower in the 2002 than the 1988 cohort. Furthermore, mean serum GA and mean serum GA/HbA1c were significantly lower in the 2012 than the 2002 cohort (these values were not assessed in the 1988 cohort). Use of lipid-modifying agents was higher in the 2012 cohort than the 2002 cohort, and the mean value of serum total cholesterol decreased over time. The frequency of electrocardiogram abnormalities and history of stroke remained unchanged from the 1988 to the 2002 cohort, followed by a significant/marginal decrease from the 2002 to the 2012 cohort. The frequency of smoking habits decreased with time, whereas alcohol intake showed the inverse trend. The frequency of regular exercise slightly decreased from the 1988 to the 2002 cohort but then increased through the 2012 cohort, while the frequency of sedentariness increased from the 1988 to the 2002 cohort and then significantly decreased to the 2012 cohort.

### Trends in the 5-year survival rate of dementia over time

**Fig. 4:**
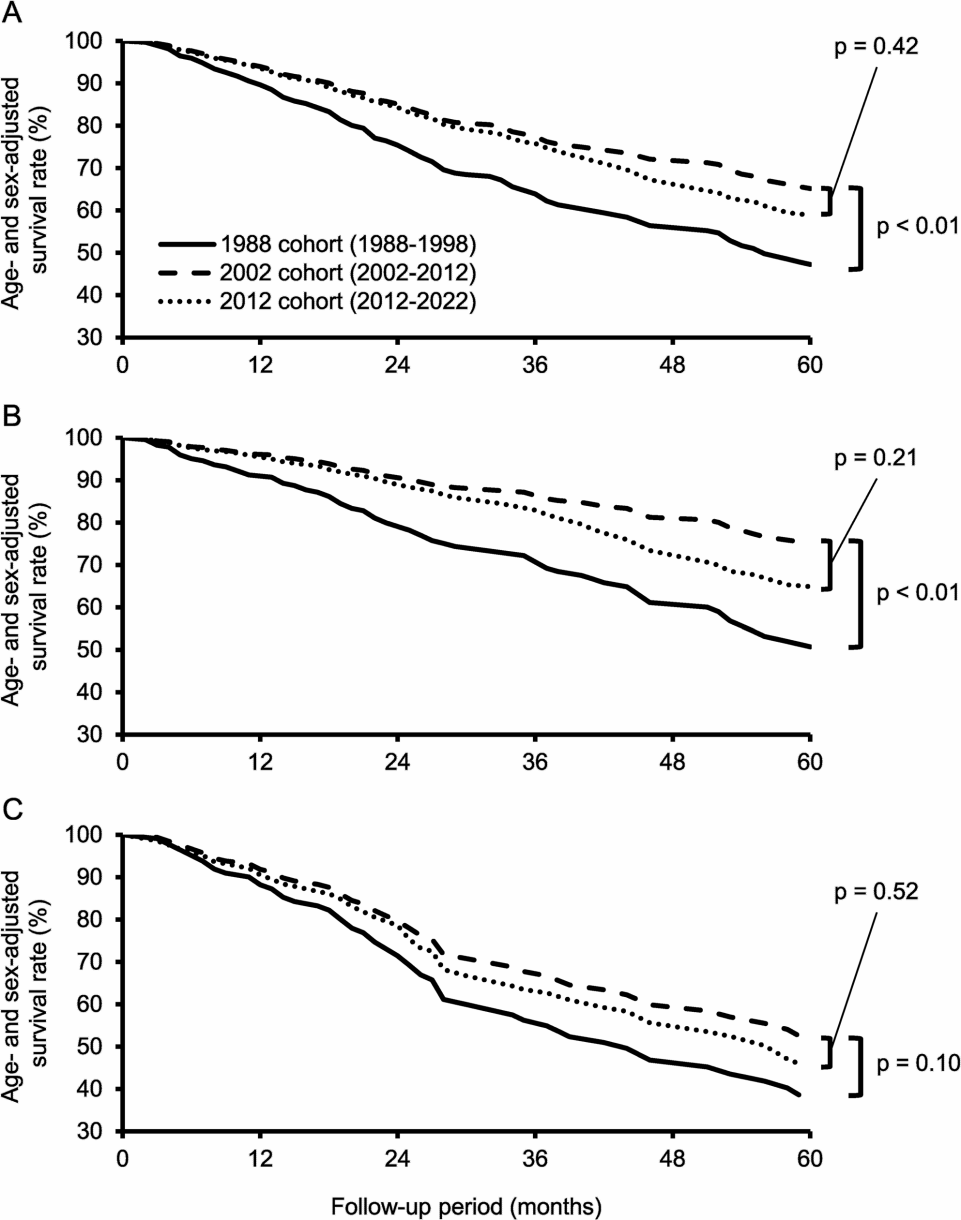
Comparison of the adjusted 5-year survival curves after incident dementia among the three cohorts. **A** All-cause dementia. **B **Alzheimer’s disease. **C **Vascular dementia Each cohort’s 5-year survival curve was adjusted for age and sex

Figure 4 shows the age- and sex-adjusted survival rate over the 5 years after dementia onset. Compared to the 1988 cohort, the 5-year survival rates for all-cause dementia and AD were significantly improved in the 2002 cohort (47.3% to 65.2% for all-cause dementia; 50.7% to 75.1% for AD; all *p* < 0.01). A similar improvement in 5-year survival was observed for VaD between the 1988 and 2002 cohorts (38.6% to 52.6%; *p* = 0.10). On the other hand, there was no significant difference in the 5-year survival rate for all-cause dementia, AD, or VaD between the 2002 and 2012 cohorts (65.2% vs. 58.9% for all-cause dementia, *p* = 0.42; 75.1% vs. 64.9% for AD, *p* = 0.21; 52.6% vs. 46.0% for VaD, *p* = 0.52). When sex-stratified analysis of survival rates was conducted, similar trends were observed for both sexes, with no evidence of heterogeneity between them (Fig. e-2).

### Trends in all-cause mortality over time

The age- and sex-adjusted all-cause mortality of survey participants was significantly decreased in the 2002 vs. the 1988 cohort (*p* < 0.01, Table e-5), but did not differ significantly between the 2002 and 2012 cohorts (Table e-6). When stratified by 5-year age group, there was a significant/marginal decrease in the all-cause mortality of participants aged 65–84 years from the 1988 to the 2002 cohort, but no significant differences were observed between the 2002 and 2012 cohorts for any age group except 85–89 years. We also compared 5-year mortality between incident dementia cases and participants without incident dementia who were randomly selected and matched for age and sex among the three cohorts. In all cohorts, the all-cause mortality of dementia cases was significantly higher than that of controls (all *p* < 0.01) (Table e-7).

## Discussion

The present study demonstrated that the age-standardized prevalence of dementia increased significantly from the 1985 to the 2012 cohort, and then showed a significant decreasing trend from 2012 to 2022. The age- and sex-adjusted incidence of dementia increased significantly from 1988 to 2002, followed by a significant decrease from 2002 to 2012. Moreover, the 5-year survival rate of dementia improved from the 1988 to the 2002 cohort and then plateaued from the 2002 to the 2012 cohort. These results may be at least partly attributable to advances in medical technology and changes in the healthcare system, as well as to modification of risk factors for dementia and promotion of healthy lifestyle behaviors.

In the present study, the prevalence of dementia in a Japanese population greatly increased from 1985 to 2012, and then began to decline after 2012. Among participants aged ≥ 85 years, the prevalence of dementia decreased from 1985 until 1998 and then increased until 2012. Although the exact reason for this trend is unclear, insufficient midlife control of vascular risk factors prior to the 1980 s may have contributed to the higher prevalence in the 1985 survey (Fig. 1), which was largely due to VaD. Subsequent improvements in blood pressure management and healthier lifestyles likely contributed to the decline in VaD prevalence among these age groups (Fig. 1). In contrast, reductions in mortality rates since the 1990 s (Table e-5) have resulted in increased longevity, thereby potentially contributing to the rising number of individuals living with dementia, particularly AD. There have been multiple population-based epidemiological studies examining trends in dementia prevalence up to around 2015. Several of these reported stable or decreasing trends in dementia prevalence in the United States [8–10] and the United Kingdom [11]. In contrast, other studies conducted in China[2], Japan[3], South Korea[4], and France [5] reported increasing trends in dementia prevalence. These results suggest that, up to around 2015, Asian countries exhibited an increasing trend in dementia prevalence, while Western countries showed a stable or declining trend. On the other hand, up to around 2020, two studies reported a stable or decreasing trend in dementia prevalence in the United States [12] and China[13], in agreement with the present study, whereas two studies in China [6] and Canada [7] indicated an increasing trend. Epidemiological studies based on medical records, primarily in Western countries, have also shown inconsistent findings regarding trends in prevalence of dementia: some have reported a decreasing or stable trend[18, 25, 39], while others observed an increasing trend [40–42]. These inconsistencies may be attributed to differences in study methodologies, including variations in the target populations, participation rates, and diagnostic criteria for dementia. Collectively, the data suggest that stable or declining trends in the prevalence of dementia, which until recently were mainly observed in Western countries, may have also emerged in Asian countries, including Japan.

In the present study, we found increasing trends in the incidence of all-cause dementia and AD from the 1988 to the 2002 cohort, whereas decreasing trends were seen from the 2002 to the 2012 cohort. There were thus inconsistent findings for the trends in dementia, especially up to around 2015. The Alzheimer Cohort Consortium, which includes seven cohorts from Western countries[14], and studies from the United States[8, 15], Nigeria[15], Sweden[16], France[17], Germany[18], the United Kingdom[19], and the Netherlands[20], reported decreasing or stable trends in dementia incidence. In contrast, reports from China[2], Taiwan[22], and South Korea[4], and a previous report by our group[21], indicate an increasing trend in dementia incidence, suggesting opposing patterns in the incidence of dementia between Western and Asian countries up to 2015. More recently, however, a report from China indicated that the previously observed increasing trend in dementia incidence slowed and then plateaued for both men and women after 2016[23], in good agreement with our results. These findings suggest that trends in the incidence of dementia in Asian countries, including Japan, are beginning to stabilize or decline, much as in Western countries.

In our earlier study, we discussed possible reasons for the increasing trends in dementia incidence from the 1988 to the 2002 cohort [21]—i.e., a decrease in the competing risk of premature death, and the westernization of lifestyle and attendant increase in metabolic risk factors. However, the present study revealed that dementia incidence declined between the 2002 and 2012 cohorts. Although the precise reason for this decline is unclear, recent epidemiological studies highlighted that prevention or good control of vascular risk factors could contribute to a reduction in the risk of incident dementia [43, 44]. In Japan, nationwide public health initiatives have long focused on reducing blood pressure and metabolic risk factors to prevent stroke and cardiovascular disease. Since the 1980 s, population-wide campaigns (e.g., the “<10 g/day salt reduction” movement) and systematic health check-ups, and since 2008, metabolic syndrome prevention programs, have contributed to improved vascular risk profiles and a decline in incidence of stroke and cardiovascular disease [45, 46]. In addition, consistent with national policies, the town of Hisayama has continued to provide intensive health guidance by public health nurses during the annual health check-up. All of these measures likely contributed to the decrease in dementia incidence observed in this study. When we analyzed the data from regular health check-ups conducted on Hisayama residents aged ≥ 65 years from 2012 to 2023, the mean systolic blood pressure significantly declined with time, while the use of antihypertensive agents remained high (Table e-8). However, the frequencies of diabetes mellitus and the use of glucose-lowering agents, particularly oral hypoglycemic agents, increased over time. In addition, the mean baseline values of serum GA and GA/HbA1c, which are considered more sensitive to glycemic variability than HbA1c, were lower in the 2012 cohort than the 2002 cohort among participants with diabetes mellitus. This may be due to recent advances in glucose-lowering agents, especially the availability in Japan of dipeptidyl peptidase-4 since 2009, glucagon-like peptide-1 receptor agonists since 2010, and sodium glucose co-transporter 2 inhibitors since 2014. In addition to these improvements to the management of lifestyle-related diseases, the frequencies of low education, history of stroke, leanness, and sedentariness decreased over time, the frequency of smoking remained low, and the frequency of regular exercise increased. Taken together, these facts suggest that prevention and proper management of lifestyle-related diseases, along with healthy lifestyle and behavior modifications, may have contributed to the proper management of vascular risk factors, which in turn could have reduced the incidence of dementia. In support of this chain of events, the age of dementia onset in our study was significantly higher in the 2012 cohort than the 2002 cohort, indicating a delay in the onset of dementia. Furthermore, the Japanese government enacted the “Basic Act on Dementia” on January 1, 2024, to strengthen national efforts against dementia; this initiative may help further advance efforts to reduce dementia risk.

Dementia is a major cause of death among older adults[47], and this association did not change from the 1988 to the 2012 cohort in the present study. However, regarding the survival rate of incident dementia cases, we found that the age- and sex-adjusted 5-year survival rate of dementia significantly improved from the 1988 to the 2002 cohort, while no secular change was observed from the 2002 to the 2012 cohort. These trends may be attributed to advances in medical technology, Japan’s medical policies, and the public long-term care insurance system implemented in Japan in 2000. Several epidemiological studies reported that the dementia-related survival rate has remained stable or increased in the United States[8, 24, 25], the Netherlands[22], Denmark[26], and China [6, 23, 27] since around 2010 or later, and all these reports support our present findings. On the other hand, a study from Germany [28] observed a decrease in dementia survival rate between 2006 and 2010. These discrepancies among studies may be due to differences in the survey year, the survey method, and the lifestyle and healthcare system of the study region.

The strengths of the present study include the long observational period; the high participation rate in each cross-sectional survey; the longitudinal population-based prospective design; the consistent methods of ascertainment and diagnosis of dementia in both cross-sectional and follow-up surveys; the almost perfect follow-up of participants; and the high frequency of use of neuroimaging and autopsy in diagnosing dementia subtypes in the follow-up survey. Several potential limitations should also be noted. First, the generalizability of our findings to the whole of Japan and to other ethnicities may be limited because the surveys were conducted in a single region. Second, the wider use of MRI in recent years could favor a diagnosis of VaD over that of AD. In our study, however, the prevalence and incidence of VaD decreased in recent years, and similar secular changes were also observed in a neuropathological study of 1,266 autopsy cases from 1986 to 2014 in our community [48]. Third, since information on social isolation was available only in selected survey years and data on head trauma, hearing impairment, and air pollution were not collected, these variables could not be evaluated. Finally, COVID-19 may have affected the mortality risk of individuals with dementia and subsequent prevalence of dementia in recent surveys. Certainly, a study from the United States [12] reported that the mortality of individuals with dementia increased from 2011 to 2021, probably due to the impact of the COVID-19 pandemic. On the other hand, we found no clear increase in mortality among all participants after 2012, and no decrease in the 5-year survival rate among those with dementia. In addition, our autopsy study was suspended beginning in 2020 due to the COVID-19 pandemic, resulting in a lower rate of evaluation by neuroimaging and/or brain autopsy in the 2012 cohort compared to the other two cohorts. Therefore, analyses regarding subtypes of dementia in the 2012 cohort may be less reliable.

## Conclusions

Decreasing trends in the prevalence and incidence of dementia were observed since 2012 in a community-dwelling Japanese older population. The decline in the incidence of dementia may be due to prevention or proper management of lifestyle-related diseases, such as hypertension and diabetes, as well as awareness and modifications of healthy lifestyle behaviors. Since the global population will continue to age in the future, greater efforts to modify the risk factors for dementia and promote healthier lifestyle behaviors are warranted to lessen the global burden of dementia.

## Supplementary Information

Supplementary Material 1: Fig. e-1. Diagram of the study design for trends in the incidence of dementia

Supplementary Material 2: Table e-1. Demographic characteristics of participants and diagnostic procedures of dementia between the 1985 and 2022 surveys. Table e-2: Comparison of adjusted incidence of dementia and its subtypes between the 1988 and 2002 cohorts. Table e-3: Comparison of adjusted incidence of dementia and its subtypes between the 2002 and 2012 cohorts. Table e-4. Crude and adjusted baseline characteristics of risk factors in the 1988, 2002 and 2012 cohorts. Table e-5: Comparison of adjusted all-cause mortality between the 1988 and 2002 cohorts. Table e-6: Comparison of adjusted all-cause mortality between the 2002 and 2012 cohorts. Table e-7. Comparison of 5-year mortality between incident dementia and without incident dementia cases in each cohort. Table e-8. Characteristics of risk factors among older participants in the 2012, 2017 and 2023 surveys

Supplementary Material 3: Fig. e-2. Adjusted 5-year survival curves for incident all-cause dementia by sex across the three cohorts

## Data Availability

The datasets used in the present study are not publicly available, because they contain confidential clinical data on the study participants. However, the data are available on reasonable request and with the permission of the Principal Investigator of the Hisayama Study, Toshiharu Ninomiya.

## Abbreviations

AD: Alzheimer’s disease
aHR: Adjusted hazard ratio
BMI: Body mass index
CI: Confidence interval
DSM-III: Diagnostic and statistical manual of mental disorders, third edition
DSM-III-R: DSM-III revised version
GA: Glycated albumin
HbA1c: Hemoglobin A1c
HDS: Hasegawa dementia scale
HDS-R: HDS revised version
HR: Hazard ratio
MMSE: Mini-mental state examination
VaD: Vascular dementia
