# Supplementary figures and images for: Thirty-seven-year trends in the prevalence, incidence, and prognosis of dementia in a Japanese community: the Hisayama study
Source: Alzheimers Res Ther. 2025 Dec 29;17:264. doi: 10.1186/s13195-025-01909-1 (PMC12751825; doi:10.1186/s13195-025-01909-1)

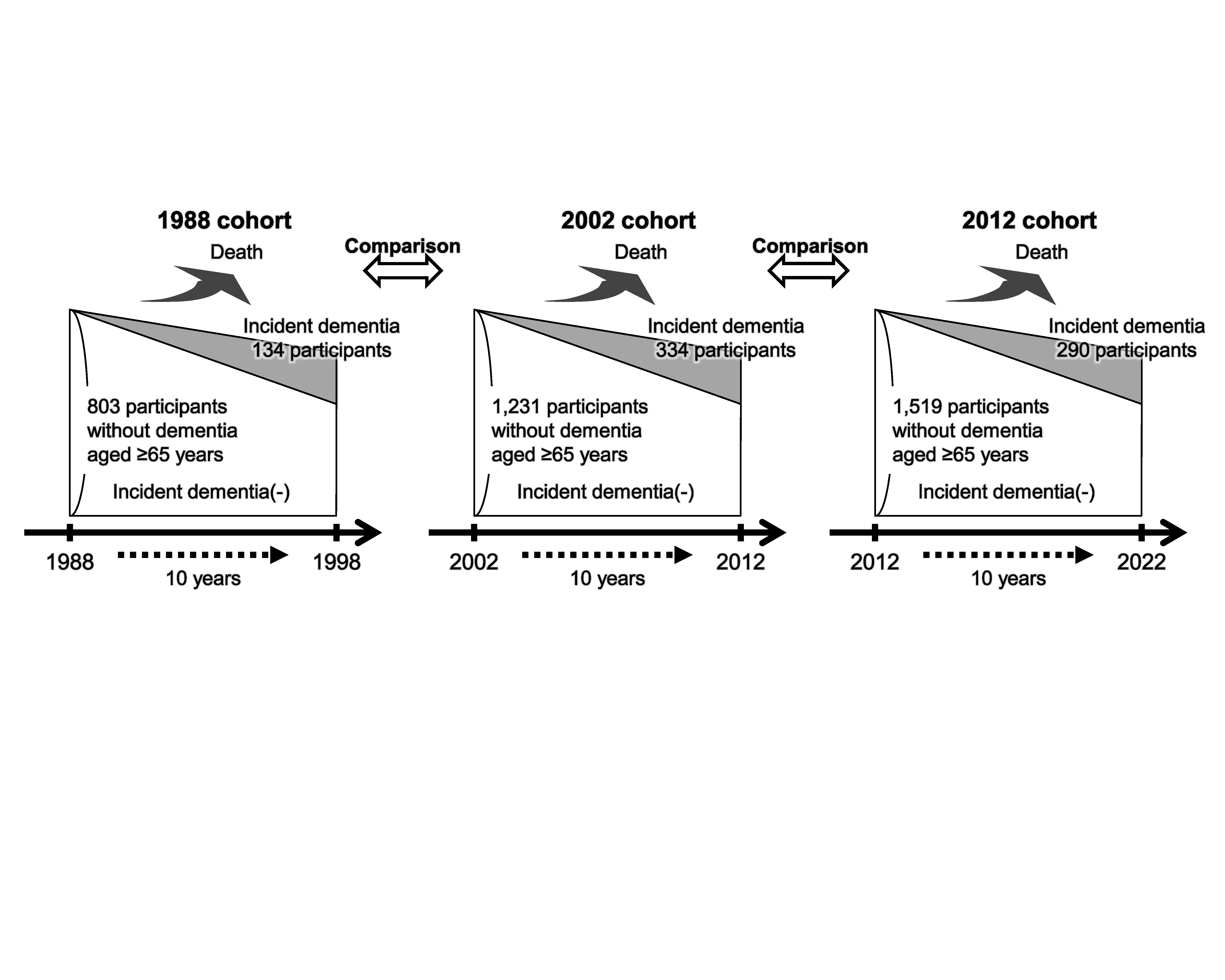

Supplement: Supplementary file 1 — Supplementary Material 1: Fig. e-1. Diagram of the study design for trends in the incidence of dementia [file 13195_2025_1909_MOESM1_ESM.tif]
