# Supplementary material for: Thirty-seven-year trends in the prevalence, incidence, and prognosis of dementia in a Japanese community: the Hisayama study
Source: Alzheimers Res Ther. 2025 Dec 29;17:264. doi: 10.1186/s13195-025-01909-1 (PMC12751825; doi:10.1186/s13195-025-01909-1)
Supplement: Supplementary file 2 — Supplementary Material 2: Table e-1. Demographic characteristics of participants and diagnostic procedures of dementia between the 1985 and 2022 surveys. Table e-2: Comparison of adjusted incidence of dementia and its subtypes between the 1988 and 2002 cohorts. Table e-3: Comparison of adjusted incidence of dementia and its subtypes between the 2002 and 2012 cohorts. Table e-4. Crude and adjusted baseline characteristics of risk factors in the 1988, 2002 and 2012 cohorts. Table e-5: Comparison of adjusted all-cause mortality between the 1988 and 2002 cohorts. Table e-6: Comparison of adjusted all-cause mortality between the 2002 and 2012 cohorts. Table e-7. Comparison of 5-year mortality between incident dementia and without incident dementia cases in each cohort. Table e-8. Characteristics of risk factors among older participants in the 2012, 2017 and 2023 surveys [file 13195_2025_1909_MOESM2_ESM.docx]

| Table e-1. Demographic characteristics of participants and diagnostic procedures of dementia between the 1985 and 2022 surveys. | | | | | | | | | | | | | | |
| --- | --- | --- | --- | --- | --- | --- | --- | --- | --- | --- | --- | --- | --- | --- |
|  |  | Yeas of survey | | | | | | | | | | | | |
|  |  | 1985  (n = 887) |  | 1992  (n = 1,189) |  | 1998  (n = 1,437) |  | 2005  (n = 1,566) |  | 2012  (n = 1,906) |  | 2017  (n = 2,202) |  | 2022  (n = 2,302) |
| Age, years, mean (SD) |  | 73.6 (6.4) |  | 74.2 (6.9) |  | 74.7 (7.2) |  | 75.9 (7.4) |  | 76.2 (7.8) |  | 76.0 (8.1) |  | 76.8 (7.8) |
| Women, % |  | 60.2 |  | 60.1 |  | 60.3 |  | 60.9 |  | 59.0 |  | 57.7 |  | 57.5 |
| Participation rate, % |  | 94.6 |  | 96.6 |  | 99.7 |  | 91.5 |  | 93.6 |  | 94.1 |  | 95.0 |
| Neuropsychological test |  | HDS |  | HDS  HDS-R  MMSE |  | HDS-R |  | HDS-R  MMSE |  | HDS-R  MMSE |  | MMSE |  | MMSE |
| Diagnosis of dementia |  | DSM-III |  | DSM-III-R |  | DSM-III-R |  | DSM-III-R |  | DSM-III-R |  | DSM-III-R |  | DSM-III-R |
| HDS = Hasegawa’s Dementia Rating Scale; HDS-R = HDS, revised version; MMSE = Mini-Mental State Examination;  DSM-III = Diagnostic and Statistical Manual of Mental Disorders, third edition;  DSM-III-R = Diagnostic and Statistical Manual of Mental Disorders, revised third edition;  SD = standard deviation. | | | | | | | | | | | | | | |

| Table e-2. Comparison of adjusted incidence of dementia and its subtypes between the 1988 and 2002 cohorts. | | | | | | | | | |
| --- | --- | --- | --- | --- | --- | --- | --- | --- | --- |
|  | 1988 cohort (1988–1998) | | |  | 2002 cohort (2002–2012) | | |  |  |
|  | Person- years at risk | No. of events | Adjusted incidence (per 1,000 preson-years and its 95% CI) |  | Person- years at risk | No. of events | Adjusted incidence (per 1,000 preson-years and its 95% CI) |  | Adjusted hazard ratio  (vs 1988 cohort) |
|  |  |  |  |  |  |  |  |  |  |
|  |  |  |  |  |  |  |  |  |  |
| All-cause dementia |  |  |  |  |  |  |  |  |  |
| Total ^a^ | 6,097 | 134 | 25.9 (21.7–30.9) |  | 9,383 | 334 | 41.6 (37.0–46.1) |  | 1.68 (1.38–2.06) |
| Men ^b^ | 2,252 | 40 | 21.6 (14.5–28.6) |  | 3,993 | 114 | 34.9 (28.0–41.7) |  | 1.71 (1.19–2.45) |
| Women ^b^ | 3,845 | 94 | 28.7 (22.8–34.7) |  | 5,390 | 220 | 45.9 (39.7–52.0) |  | 1.68 (1.32–2.14) |
| Alzheimer’s disease |  |  |  |  |  |  |  |  |  |
| Total ^a^ | 6,097 | 73 | 14.6 (11.6–18.9) |  | 9,383 | 222 | 28.2 (24.2–31.7) |  | 2.07 (1.59–2.70) |
| Men ^b^ | 2,252 | 12 | 6.6 (2.7–10.6) |  | 3,993 | 61 | 19.9 (14.6–25.3) |  | 3.03 (1.63–5.64) |
| Women ^b^ | 3,845 | 61 | 19.7 (14.6–24.7) |  | 5,390 | 161 | 33.5 (28.2–38.7) |  | 1.89 (1.41–2.54) |
| Vascular dementia |  |  |  |  |  |  |  |  |  |
| Total ^a^ | 6,097 | 48 | 9.3 (6.4–11.7) |  | 9,383 | 87 | 10.6 (8.4–12.9) |  | 1.18 (0.83–1.69) |
| Men ^b^ | 2,252 | 23 | 12.2 (6.9–17.5) |  | 3,993 | 37 | 10.7 (6.9–14.4) |  | 0.95 (0.57–1.60) |
| Women ^b^ | 3,845 | 25 | 7.4 (4.4–10.4) |  | 5,390 | 50 | 10.6 (7.6–13.6) |  | 1.40 (0.87–2.27) |
| Other/unclassified dementia |  |  |  |  |  |  |  |  |  |
| Total ^a^ | 6,097 | 20 | 3.8 (2.0–5.3) |  | 9,383 | 40 | 4.7 (3.3–6.3) |  | 1.32 (0.77–2.27) |
| Men ^b^ | 2,252 | 7 | 4.3 (0.9–7.8) |  | 3,993 | 19 | 5.2 (2.7–7.7) |  | 1.60 (0.67–3.81) |
| Women ^b^ | 3,845 | 13 | 3.5 (1.5–5.4) |  | 5,390 | 21 | 4.4 (2.5–6.3) |  | 1.17 (0.58–2.23) |
| CI = confidence interval.  ^a^ Adjusted for age and sex  ^b^ Adjusted for age alone. | | | | | | | | | |

| Table e-3. Comparison of adjusted incidence of dementia and its subtypes between the 2002 and 2012 cohorts. | | | | | | | | | |
| --- | --- | --- | --- | --- | --- | --- | --- | --- | --- |
|  | 2002 cohort (2002–2012) | | |  | 2012 cohort (2012–2022) | | |  |  |
|  | Person- years at risk | No. of events | Adjusted incidence (per 1,000 preson-years and its 95% CI) |  | Person- years at risk | No. of events | Adjusted incidence (per 1,000 preson-years and its 95% CI) |  | Adjusted hazard ratio  (vs 2002 cohort) |
|  |  |  |  |  |  |  |  |  |  |
|  |  |  |  |  |  |  |  |  |  |
| All-cause dementia |  |  |  |  |  |  |  |  |  |
| Total ^a^ | 9,383 | 334 | 41.6 (37.0–46.1) |  | 11,849 | 290 | 28.0 (24.7–31.3) |  | 0.60 (0.51–0.70) |
| Men ^b^ | 3,993 | 114 | 34.9 (28.0–41.7) |  | 4,962 | 111 | 25.9 (20.8–30.9) |  | 0.66 (0.51–0.86) |
| Women ^b^ | 5,390 | 220 | 45.9 (39.7–52.0) |  | 6,887 | 179 | 29.6 (25.2–34.0) |  | 0.56 (0.46–0.69) |
| Alzheimer’s disease |  |  |  |  |  |  |  |  |  |
| Total ^a^ | 9,383 | 222 | 28.2 (24.2–31.7) |  | 11,849 | 210 | 20.5 (17.7–23.3) |  | 0.65 (0.54–0.79) |
| Men ^b^ | 3,993 | 61 | 19.9 (14.6–25.3) |  | 4,962 | 74 | 17.8 (13.5–22.1) |  | 0.79 (0.57–1.12) |
| Women ^b^ | 5,390 | 161 | 33.5 (28.2–38.7) |  | 6,887 | 136 | 22.5 (18.7–26.4) |  | 0.59 (0.47–0.74) |
| Vascular dementia |  |  |  |  |  |  |  |  |  |
| Total ^a^ | 9,383 | 87 | 10.6 (8.4–12.9) |  | 11,849 | 51 | 4.7 (3.4–6.0) |  | 0.41 (0.29–0.58) |
| Men ^b^ | 3,993 | 37 | 10.7 (6.9–14.4) |  | 4,962 | 28 | 6.0 (3.7–8.2) |  | 0.54 (0.33–0.89) |
| Women ^b^ | 5,390 | 50 | 10.6 (7.6–13.6) |  | 6,887 | 23 | 3.7 (2.2–5.3) |  | 0.32 (0.20–0.53) |
| Other/unclassified dementia |  |  |  |  |  |  |  |  |  |
| Total ^a^ | 9,383 | 40 | 4.7 (3.3–6.3) |  | 11,849 | 45 | 4.3 (3.0–5.6) |  | 0.80 (0.52–1.22) |
| Men ^b^ | 3,993 | 19 | 5.2 (2.7–7.7) |  | 4,962 | 19 | 4.2 (2.2–6.3) |  | 0.74 (0.39–1.40) |
| Women ^b^ | 5,390 | 21 | 4.4 (2.5–6.3) |  | 6,887 | 26 | 4.4 (2.7–6.1) |  | 0.85 (0.48–1.51) |
| CI = confidence interval.  ^a^ Adjusted for age and sex  ^b^ Adjusted for age alone. | | | | | | | | | |

| Table e-4. Crude and adjusted baseline characteristics of risk factors in the 1988, 2002 and 2012 cohorts. | | | | | | | | |
| --- | --- | --- | --- | --- | --- | --- | --- | --- |
|  | 1988 cohort (n = 803) | |  | 2002 cohort (n = 1,231) | |  | 2012 cohort (n = 1,519) | |
|  | Crude | Adjusted |  | Crude | Adjusted |  | Crude | Adjusted |
| Age, years | 74.1 (6.1) | 74.1 (6.4) |  | 73.7 (6.6) | 73.7 (6.4) |  | 74.5 (6.7) ^*^ | 74.5 (6.5) ^*^ |
| Men, % | 39.0 ^†^ | 39.0 ^†^ |  | 43.0 | 43.0 |  | 43.0 | 43.0 |
| Education ≤9 years, % | 73.3 ^*^ | 73.5 ^*^ |  | 57.9 | 58.1 |  | 39.6 ^*^ | 38.9 ^*^ |
| Systolic blood pressure, mmHg | 144 (25) ^*^ | 144 (21) ^*^ |  | 139 (20) | 139 (21) |  | 135 (19) ^*^ | 134 (22) ^*^ |
| Diastolic blood pressure, mmHg | 76 (11) ^*^ | 75 (11) ^*^ |  | 79 (11) | 79 (11) |  | 77 (11) ^*^ | 76 (12) ^*^ |
| Hypertension, % | 62.0 | 62.1 |  | 62.7 | 62.9 |  | 71.5 ^*^ | 72.3 ^*^ |
| Use of antihypertensive agents, % | 28.8 ^*^ | 28.6 ^*^ |  | 38.7 | 38.6 |  | 56.0 ^*^ | 55.9 ^*^ |
| Body mass index, kg/m2 | 22.0 (3.2) ^*^ | 21.9 (3.3) ^*^ |  | 22.9 (3.3) | 22.8 (3.3) |  | 23.1 (3.4) ^†^ | 23.1 (3.6) ^*^ |
| Leanness, % | 14.0 ^*^ | 13.1 ^*^ |  | 8.9 | 8.3 |  | 7.8 | 7.4 |
| Obesity, % | 17.4 ^*^ | 16.7 ^*^ |  | 25.1 | 24.6 |  | 26.5 | 26.4 |
| Diabetes mellitus, % | 13.6 ^*^ | 13.4 ^*^ |  | 22.9 | 22.4 |  | 24.3 | 23.9 |
| Use of glucose-lowering agents, % | 4.9 ^*^ | 4.9 ^*^ |  | 8.9 | 8.8 |  | 13.4 ^*^ | 13.2 ^*^ |
| Oral hypoglycemic agents, % | 4.4 ^*^ | 4.4 ^*^ |  | 7.8 | 7.7 |  | 12.8 ^*^ | 12.6 ^*^ |
| Insulin injection, % | 0.8 ^*^ | 0.7 ^†^ |  | 1.9 | 1.8 |  | 1.8 | 1.8 |
| Serum total cholesterol, mmol/L | 5.4 (1.1) ^*^ | 5.3 (0.9) ^*^ |  | 5.2 (0.9) | 5.2 (0.9) |  | 5.1 (0.9) ^*^ | 5.1 (1.0) ^*^ |
| Hypercholesterolemia, % | 35.2 ^*^ | 33.2 ^*^ |  | 39.8 | 39.3 |  | 48.9 ^*^ | 49.3 ^*^ |
| Use of lipid-modifying agents, % | NA | NA |  | 15.8 | 15.4 |  | 34.7 ^*^ | 34.4 ^*^ |
| Electrocardiogram abnormalities, % | 23.0 | 23.0 |  | 23.3 | 23.0 |  | 16.8 ^*^ | 16.4 ^*^ |
| History of stroke, % | 6.2 | 6.0 |  | 6.2 | 5.9 |  | 4.7 ^†^ | 4.5 ^†^ |
| Current smoking habits, % | 19.8 ^*^ | 15.7 ^*^ |  | 13.2 | 8.8 |  | 8.6 ^*^ | 5.6 ^*^ |
| Current alcohol intake, % | 22.0 ^*^ | 17.0 ^*^ |  | 32.9 | 26.8 |  | 40.4 ^*^ | 37.8 ^*^ |
| Regular exercise, % | 16.7 | 16.6 |  | 14.2 | 14.0 |  | 19.1 ^*^ | 18.9 ^*^ |
| Sedentariness, % | 7.2 ^*^ | 5.1 ^*^ |  | 11.2 | 8.4 |  | 4.4 ^*^ | 2.7 ^*^ |
|  |  |  |  |  |  |  |  |  |
| ***In participants with diabetes mellitus*** | 1988 cohort (n = 109) | |  | 2002 cohort (n = 281) | |  | 2012 cohort (n = 329) | |
| HbA1c, % | 7.1 (1.3) ^*^ | 7.1 (1.1) ^*^ |  | 6.5 (1.3) | 6.5 (1.1) |  | 6.6 (0.8) | 6.6 (1.1) |
| Serum GA, % | NA | NA |  | 19.6 (5.2) | 19.7 (4.4) |  | 18.8 (3.5) ^*^ | 18.7 (4.3) ^*^ |
| Serum GA/HbA1c | NA | NA |  | 3.0 (0.5) | 3.0 (0.4) |  | 2.9 (0.4) ^*^ | 2.8 (0.4) ^*^ |
| ^*^ p < 0.05 vs. 2002 cohort; ^†^ p < 0.10 vs. 2002 cohort. HbA1c =hemoglobin A1c; GA=glycated albumin; NA=not assessed All values are given as the mean (standard deviation) or as a percent. Mean age was sex-adjusted. Percentage of men was age-adjusted. | | | | | | | | |

| Table e-5. Comparison of adjusted all-cause mortality between the 1988 and 2002 cohorts. | | | | | | | | | |
| --- | --- | --- | --- | --- | --- | --- | --- | --- | --- |
|  | 1988 cohort (1988–1998) | | |  | 2002 cohort (2002–2012) | | |  |  |
| Age  group,  years | Person- years at risk | No. of events | Adjusted mortality (per 1,000 preson-years and its 95% CI) |  | Person- years at risk | No. of events | Adjusted mortality (per 1,000 preson-years and its 95% CI) |  | Adjusted hazard ratio (vs 1988 cohort) |
| All ^a^ | 6478 | 288 | 50.6 (42.9–54.2) |  | 10365 | 335 | 36.2 (31.8–39.6) |  | 0.68 (0.58–0.79) ^**^ |
| 65–69 ^b^ | 2017 | 37 | 19.0 (12.7–25.3) |  | 3526 | 41 | 12.0 (8.3–15.6) |  | 0.64 (0.41–0.99) ^*^ |
| 70–74 ^b^ | 2032 | 60 | 32.5 (24.1–40.9) |  | 2990 | 67 | 23.9 (18.1–29.8) |  | 0.75 (0.53–1.02) |
| 75–79 ^b^ | 1407 | 82 | 66.6 (51.3–81.8) |  | 2230 | 88 | 40.8 (32.2–49.4) |  | 0.61 (0.45–0.83) ^**^ |
| 80–84 ^b^ | 731 | 70 | 108.9 (81.9–135.9) |  | 1085 | 64 | 70.5 (51.9–89.2) |  | 0.61 (0.43–0.85) ^**^ |
| 85–89 ^b^ | 255 | 30 | 108.1 (75.1–141.1) |  | 388 | 53 | 119.7 (117.8–121.6) |  | 1.10 (0.69–1.76) |
| 90– ^b^ | 36 | 9 | 285.7 (252.8–318.7) |  | 146 | 22 | 165.4 (164.0–166.9) |  | 0.45 (0.20–1.02) |
| ^a^ Age- and sex-adjusted. ^b^ Sex-adjusted. CI = confidence interval. ^*^ p <0.05, ^**^ p <0.01 vs. the 1988 cohort. | | | | | | | | | |

| Table e-6. Comparison of adjusted all-cause mortality between the 2002 and 2012 cohorts. | | | | | | | | | |
| --- | --- | --- | --- | --- | --- | --- | --- | --- | --- |
|  | 2002 cohort (2002–2012) | | |  | 2012 cohort (2012–2022) | | |  |  |
| Age  group,  years | Person- years at risk | No. of events | Adjusted mortality (per 1,000 preson-years and its 95% CI) |  | Person- years at risk | No. of events | Adjusted mortality (per 1,000 preson-years and its 95% CI) |  | Adjusted hazard ratio (vs 2002 cohort) |
| All ^a^ | 10365 | 335 | 36.2 (31.8–39.6) |  | 13591 | 399 | 33.6 (30.3–37.0) |  | 0.91 (0.79–1.06) |
| 65–69 ^b^ | 3526 | 41 | 12.0 (8.3–15.6) |  | 4063 | 44 | 11.4 (10.5–12.4) |  | 1.09 (0.71–1.68) |
| 70–74 ^b^ | 2990 | 67 | 23.9 (18.1–29.8) |  | 3938 | 73 | 19.6 (18.3–20.8) |  | 0.87 (0.62–1.21) |
| 75–79 ^b^ | 2230 | 88 | 40.8 (32.2–49.4) |  | 2907 | 96 | 35.0 (33.5–36.5) |  | 0.95 (0.71–1.28) |
| 80–84 ^b^ | 1085 | 64 | 70.5 (51.9–89.2) |  | 1726 | 97 | 59.9 (58.2–61.5) |  | 1.09 (0.78–1.52) |
| 85–89 ^b^ | 388 | 53 | 119.7 (117.8–121.6) |  | 745 | 62 | 89.3 (87.8–90.8) |  | 0.66 (0.45–0.96) ^*^ |
| 90– ^b^ | 146 | 22 | 165.4 (164.0–166.9) |  | 212 | 27 | 139.2 (137.9–140.4) |  | 0.84 (0.48–1.48) |
| ^a^ Age- and sex-adjusted. ^b^ Sex-adjusted. CI = confidence interval. ^*^ p <0.05 vs. 2002 cohort. | | | | | | | | | |

| Table e-7. Comparison of 5-year mortality between incident dementia and without incident dementia cases in each cohort. | | | | | |
| --- | --- | --- | --- | --- | --- |
|  | Population at risk | Number of events | Hazard ratio (per 1,000 preson-years and its 95% CI) | p |  |
| 1988 cohort |  |  |  |  |  |
| Without incident dementia | 102 | 16 | 1.00 (reference) |  |  |
| Incident dementia | 102 | 34 | 2.35 (1.30–4.25) | <0.01 |  |
|  |  |  |  |  |  |
| 2002 cohort |  |  |  |  |  |
| Without incident dementia | 267 | 42 | 1.00 (reference) |  |  |
| Incident dementia | 267 | 69 | 1.76 (1.20–2.59) | <0.01 |  |
|  |  |  |  |  |  |
| 2012 cohort |  |  |  |  |  |
| Without incident dementia | 232 | 45 | 1.00 (reference) |  |  |
| Incident dementia | 232 | 82 | 1.92 (1.33–2.76) | <0.01 |  |
|  |  |  |  |  |  |
| p for heterogeneity between the 1988 and the 2002 cohorts | | |  | 0.49 |  |
| p for heterogeneity between the 2002 and the 2012 cohorts | | |  | 0.89 |  |
| CI = confidence interval.  Participants without incident dementia were randomly selected, matched by age and sex to participants with incident dementia | | | | | |

| Table e-8. Characteristics of risk factors among older participants in the 2012, 2017 and 2023 surveys. | | | | | | | | | | | |
| --- | --- | --- | --- | --- | --- | --- | --- | --- | --- | --- | --- |
|  | 2012 (n = 1,711) | |  | 2017 (n = 1,901) | |  | 2023 (n = 1,394) | |  | p for trend | |
|  | Crude | Adjusted |  | Crude | Adjusted |  | Crude | Adjusted |  | Crude | Adjusted |
| Age, years | 75.5 (7.7) | 75.5 (7.4) |  | 75.2 (7.7) | 75.2 (7.4) |  | 74.8 (6.5) ^*^ | 74.8 (7.4) ^*^ |  | <0.01 | <0.01 |
| Men, % | 42.4 | 42.6 |  | 43.2 | 43.2 |  | 45.8 ^†^ | 45.6 ^†^ |  | 0.06 | 0.098 |
| Education ≤9 years, % | 41.0 | 40.2 |  | 30.7 ^*^ | 29.6 ^*^ |  | 17.7 ^*^ | 16.5 ^*^ |  | <0.01 | <0.01 |
| Systolic blood pressure, mmHg | 136 (20) | 135 (19) |  | 132 (20) ^*^ | 132 (19) ^*^ |  | 129 (18) ^*^ | 129 (19) ^*^ |  | <0.01 | <0.01 |
| Diastolic blood pressure, mmHg | 76 (11) | 76 (11) |  | 71 (12) ^*^ | 71 (11) ^*^ |  | 70 (11) ^*^ | 70 (11) ^*^ |  | <0.01 | <0.01 |
| Hypertension, % | 71.7 | 72.3 |  | 67.6 ^*^ | 68.5 ^*^ |  | 67.6 ^*^ | 68.8 ^*^ |  | 0.01 | 0.03 |
| Use of antihypertensive agents, % | 55.3 | 55.2 |  | 53.7 | 53.8 |  | 55.9 | 56.5 |  | 0.80 | 0.53 |
| Body mass index, kg/m^2^ | 22.9 (3.5) | 22.9 (3.5) |  | 23.0 (3.6) | 23.0 (3.5) |  | 23.3 (3.6) ^*^ | 23.2 (3.5) ^*^ |  | <0.01 | <0.01 |
| Leanness, % | 10.1 | 8.9 |  | 9.3 | 8.3 |  | 6.9 ^*^ | 6.5 ^*^ |  | <0.01 | 0.02 |
| Obesity, % | 25.0 | 24.9 |  | 27.1 | 26.8 |  | 27.3 | 26.7 |  | 0.13 | 0.22 |
| Diabetes mellitus, % | 23.7 | 23.3 |  | 24.7 | 24.2 |  | 26.7 ^†^ | 25.7 |  | 0.06 | 0.12 |
| Use of glucose-lowering agents, % | 13.3 | 12.9 |  | 14.6 | 14.1 |  | 16.0 ^*^ | 15.2 ^†^ |  | 0.04 | 0.06 |
| Oral hypoglycemic agents, % | 12.7 | 12.3 |  | 14.2 | 13.7 |  | 15.9 ^*^ | 15.2 ^*^ |  | 0.01 | 0.02 |
| Insulin injection, % | 1.9 | 1.8 |  | 1.8 | 1.7 |  | 0.9 ^*^ | 0.9 ^*^ |  | 0.04 | 0.03 |
| Serum total cholesterol, mmol/L | 5.1 (1.0) | 5.1 (0.9) |  | 5.2 (1.0) ^*^ | 5.2 (0.9) ^*^ |  | 5.4 (1.0) ^*^ | 5.4 (0.9) ^*^ |  | <0.01 | <0.01 |
| Hypercholesterolemia, % | 52.8 | 52.9 |  | 60.2 ^*^ | 60.8 ^*^ |  | 67.4 ^*^ | 68.9 ^*^ |  | <0.01 | <0.01 |
| Use of lipid-modifying agents, % | 33.0 | 32.6 |  | 34.6 | 34.4 |  | 39.6 ^*^ | 39.8 ^*^ |  | <0.01 | <0.01 |
| Electrocardiogram abnormalities, % | 17.7 | 17.2 |  | 16.4 | 16.0 |  | 13.6 ^*^ | 13.4 ^*^ |  | <0.01 | <0.01 |
| History of stroke, % | 7.7 | 7.0 |  | 6.4 | 5.8 |  | 4.8 ^*^ | 4.4 ^*^ |  | <0.01 | <0.01 |
| Current smoking habits, % | 8.4 | 5.3 |  | 8.4 | 5.2 |  | 8.8 | 5.4 |  | 0.71 | 0.95 |
| Current alcohol intake, % | 37.1 | 35.2 |  | 40.5 ^*^ | 38.6 ^†^ |  | 46.3 ^*^ | 44.0 ^*^ |  | <0.01 | <0.01 |
| Regular exercise, % | 16.2 | 15.9 |  | 18.4 ^†^ | 17.9 |  | 22.9 ^*^ | 22.1 ^*^ |  | <0.01 | <0.01 |
| Sedentariness, % | 12.2 | 7.3 |  | 8.8 ^*^ | 5.0 ^*^ |  | 6.8 ^*^ | 4.7 ^*^ |  | <0.01 | <0.01 |
| All participants were aged 65 years or older at the time of each survey.  ^*^ p < 0.05 vs. 2012 survey; ^†^ p < 0.10 vs. 2012 survey.  All values are given as the mean (standard deviation) or as a percent. Mean age was sex-adjusted. Percentage of men was age-adjusted. | | | | | | | | | | | |
